# Compendium on Food Crop Plants as a Platform for Pharmaceutical Protein Production

**DOI:** 10.3390/ijms23063236

**Published:** 2022-03-17

**Authors:** Aneta Gerszberg, Katarzyna Hnatuszko-Konka

**Affiliations:** Department of Molecular Biotechnology and Genetics, Faculty of Biology and Environmental Protection, University of Lodz, Banacha 12/16, 90-237 Lodz, Poland; katarzyna.hnatuszko@biol.uni.lodz.pl

**Keywords:** recombinant proteins, crop plants, molecular farming, biofactories

## Abstract

Tremendous advances in crop biotechnology related to the availability of molecular tools and methods developed for transformation and regeneration of specific plant species have been observed. As a consequence, the interest in plant molecular farming aimed at producing the desired therapeutic proteins has significantly increased. Since the middle of the 1980s, recombinant pharmaceuticals have transformed the treatment of many serious diseases and nowadays are used in all branches of medicine. The available systems of the synthesis include wild-type or modified mammalian cells, plants or plant cell cultures, insects, yeast, fungi, or bacteria. Undeniable benefits such as well-characterised breeding conditions, safety, and relatively low costs of production make plants an attractive yet competitive platform for biopharmaceutical production. Some of the vegetable plants that have edible tubers, fruits, leaves, or seeds may be desirable as inexpensive bioreactors because these organs can provide edible vaccines and thus omit the purification step of the final product. Some crucial facts in the development of plant-made pharmaceuticals are presented here in brief. Although crop systems do not require more strictly dedicated optimization of methodologies at any stages of the of biopharmaceutical production process, here we recall the complete framework of such a project, along with theoretical background. Thus, a brief review of the advantages and disadvantages of different systems, the principles for the selection of *cis* elements for the expression cassettes, and available methods of plant transformation, through to the protein recovery and purification stage, are all presented here. We also outline the achievements in the production of biopharmaceuticals in economically important crop plants and provide examples of their clinical trials and commercialization.

## 1. Introduction

The pharmaceutical market for recombinant biopharmaceuticals is enormous and is constantly growing. This is fully justified since recombinant proteins have transformed the treatment of a broad range of diseases and are used in practically all branches of medicine. To meet the needs of this demanding market, efficient and economical expression platforms for the production of therapeutic proteins are sought. Among the most commonly used production systems are bacterial, mammalian, yeast and insect cells, cell suspensions, or filamentous fungi [1]. They all have advantages and disadvantages that are briefly summarized in the subsequent section, as comparing them has already been the subject of many excellent reviews [1,2,3,4].

The concept of using plants as a platform for producing recombinant proteins emerged in the second half of the 1980s. Then, in 1986, human growth hormone was produced in tobacco cells [5], while in 1989, methods of antibody production in tobacco plants were conceived [6]. The results of the above-mentioned research resulted in a patent being issued [7]. Later, in 1990, the production of human serum albumin in tobacco and potato cells became possible [8]. The years that followed brought numerous academic studies which proved unequivocally that it is possible to produce a wide range of biopharmaceuticals in various plant platforms. Moreover, in some cases, the entire production process of plant-made pharmaceuticals (PMP) has been completed with commercialization, which will be discussed later in the publication. The subsequent significant developments in recombinant protein production in plants are shown in Table 1.

Recombinant DNA technology (recDNA technology) has made it possible to cross interspecies barriers and thus acquire new plant varieties with desirable traits. Genetic modifications of crop plants mainly concern improving their utility or resistance to changing environmental conditions [19]. Nevertheless, some studies are strictly focused on the production of therapeutic proteins in plants, including vegetables. What arouses great interest in cultivated plants as platforms for the production of therapeutic proteins is the fact that they are the basis of the human diet, and what is more, the edible tissues of these plants can be used, for example, as edible vaccines. This means that the costly step of purifying the final product (therapeutic protein) is omitted, which significantly reduces the overall cost of production. Among the key challenges for the plant oral delivery system one can name the accumulation of a stable and optimal dose of biopharmaceuticals. This requires the administration of the drug in question in the form of tissue pulp/bulk. Usually, the plant material undergoes a lyophilization process that ensures adequate content and stability. Then, the candidate for a drug, as in the case of any other substance, regardless of the administration route, must be tested and meet the Food and Drug Administration (FDA) and European Medicines Agency (EMA) criteria of preclinical and clinical trials in good laboratory, manufacturing, and clinical practice (GLP, GMP, GCP) standards. The detailed directions for human health, established by OECD are available on the institution’s website [20]. 

Initially, model plants were used for the production of recombinant proteins, as they were very well known in the field of genomics, transcriptomics, and metabolomics. Moreover, as these plants are susceptible to genetic transformation, fully optimized protocols for regeneration and cultivation under both in vitro and ex vitro conditions have been developed for them [21,22]. Nevertheless, they have several features that exclude them from their intended use in molecular farming technology. For example, the small size of *Nicotiana tabacum* or *Arabidopsis thaliana* seeds favours the leaves for the production of recombinant proteins. Unfortunately, the large amount of water they contain can promote the proteolysis process, which will preclude the extraction of the final protein product. Additionally, the extraction and purification of therapeutic proteins from tobacco is expensive due to the presence of alkaloids (e.g., nicotine) that are toxic to humans. Crops, including soybean, rapeseed, common bean, maize, wheat, and rice, seem to be free from these drawbacks [22,23,24]. Therefore, they can be successfully used as a factory for molecular farming and as a subject of biofortification, aiming at the production of crops with increased nutritional value, which can be obtained through genetic engineering [23,25]. 

## 2. Advantages and Disadvantages of Pharmaceuticals Production in Available Expression Systems

The process of developing the production of a specific therapeutic protein in any system (including crop plants) consists of several stages, each being extremely complex. First, a particular protein with the desired therapeutic activity should be selected and subjected to very precise molecular analysis. This knowledge will form the basis of optimizing the expression of this protein in the selected plant system and even in a specific cell compartment. The next step is to introduce the gene encoding the desired protein into the recipient’s genome, using the appropriate transformation method and host organism (here, the plant). It should be emphasized that it is also indispensable to estimate the expected costs of obtaining a protein product based on the work with the prototype host [26].

When planning the production process, several important factors should be considered, including production costs, market demand, and the efficiency of the production method, as well as product safety and stability. The final product synthesized in a plant should be identical in terms of biochemical/pharmacological properties to that produced with the methods used so far. Currently, the most commonly used systems for the production of recombinant proteins are cultures of genetically modified mammalian cells, insects, yeast, fungi, or bacteria [27]. Taking into account the uncomplicated synthesis technology as well as its costs, bacterial or fungal cells are one of the most popular production systems for therapeutic proteins. Nevertheless, both bacteria and fungi have some drawbacks that limit them only to the production of selected proteins. One of the main obstacles relates to the differences in the course of metabolic pathways—the process of translation, folding, and post-translational modification of proteins (e.g., glycosylation) that affects the structure of the product obtained, as well as its biological activity [22]. While in the case of mammalian or insect cells there are no such limitations, the economic reasons, such as the need to use expensive culture media, make the market price of the final product very high [27]. Furthermore, the production costs of therapeutic proteins in mammalian or insect cell systems are raised by the influence of biological factors on the behaviour of the cultures in question. These include the sensitivity of the culture to changes in physicochemical conditions. Similarly, the yeast platform, which can be used as an alternative for therapeutics synthetized in an insoluble form in bacteria, is not able to perform the desired glycosylation. This disadvantage includes the inability to provide high-mannose type N-glycosylation, typical for the cells of higher eukaryotes. Considering all the limitations of the systems being discussed and the growing market demand for therapeutic proteins, plants seem to be an interesting alternative for their production. The key advantages of plant systems are the ease of cultivation, low costs, ease of production scaling, low or lack of any risk of contamination with pathogens, the ability to carry out most post-translation modifications of proteins, and finally, lack of ethical doubts [1]. However, despite having so many advantages, plant systems also have several drawbacks: differences in protein glycosylation patterns between plants and animals can cause allergies, while pollution with secondary metabolites, pesticides, or herbicides can be harmful to people. Moreover, the long period of the plant growth can be listed as another disadvantage [1,22,28].

## 3. Molecular Tools for Therapeutic Protein Production in Crop Plants

Over the past decades a number of transformation methods facilitating the development of molecular farming technology were established and improved. The most widely used among them are particle bombardment and the *Agrobacterium* transformation method [29]. Different strategies available for the transfer of transgenes into host plant cells were reviewed and summarized in detail by Keshavareddy et al. [30].

In the process of obtaining transgenic plants, apart from selecting an appropriate method for the transformation of crop plant tissues and an effective method for selection and regeneration, constructing a gene expression cassette is an extremely important step.

**Figure 1:**
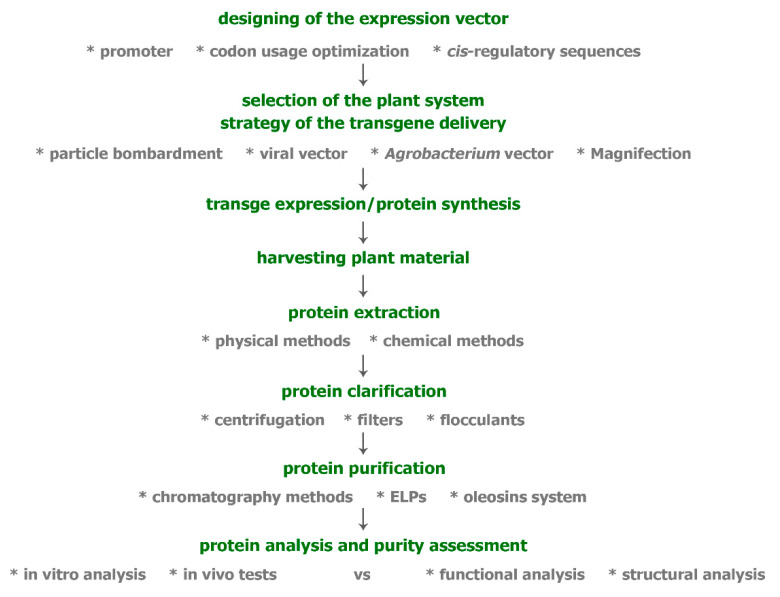
Schematic diagram of the production of biopharmaceuticals in transgenic plants.

All the issues that have arisen in the production of biopharmaceuticals in transgenic plants are summarized in a schematic diagram (Figure 1).

A well-designed and experimentally tested expression cassette will allow for achieving the optimal/satisfactory level of transgene expression in a given tissue or plant development stage or under specific environmental induction conditions. A standard expression cassette contains a transgene or transgenes under the control of the appropriate promoter and set of regulatory sequences. Expression of transgenes is dependent on the type of promoter used, which may reveal a constitutive (e.g., *Zea mays* ubiquitin promoter, *ZmUbi1*), inducible (e.g., *Rosa bourboniana* an early wound inducible promoter, *RbPCD1pro*), or tissue-specific (e.g., the wheat low-molecular-weight glutenin *promoter, LMWG1D1*) pattern of expression [31,32,33]. Due to the increasing number of sequenced plant genomes, as well as remarkable progress in sequencing technology and transcriptome analysis, the number of promoters available for plant transgenesis has increased significantly [31]. Among most frequently used are cauliflower mosaic virus 35S (CaMV35S), glutelin, zein, arcelin, E8, and actin promoters. A detailed review of promoters and *cis*-acting regulatory elements was made by Biłas et al. [34] and Ali and Kim [32]. Typically, well-known plant-derived promoters are active in transgenic plants, although they may show activity different from that observed in species they originated from. In this context, the level of transgene activity depends on the length of the promoter region selected [32]. Nevertheless, there are cases of a variable level of transgene expression activity. Hence, it seems necessary to first evaluate the activity of the selected promoter experimentally in relation to the genetic background of the plant selected for transformation. Considering this problem and the importance of determining the individual module functions of the *cis* elements, designing synthetic promoters (e.g., *mPtDrl02* promoter) seems a good solution. These types of promoters would contain functional modules that would precisely define their action, including specificity and strength [33,34]. Such an approach would allow the intended goal to be achieved—i.e., obtaining plants with the desired agronomic traits or synthesis of therapeutic proteins, biopharmaceuticals, or industrial enzymes at a high level.

Apart from a promoter, an expression cassette should include regulatory elements of a type, which depends on the host, expression time and localization, and protein application. Hence, the construct may carry the sequences for enhancers, silencers, and/or insulators. Moreover, the 5′UTR and 3′UTR sequences (untranslated region) affecting, i.a., translation process should be properly chosen. Regarding the final localization in cell compartments, short signal sequences (e.g., HDEL) can be fused to the transgene sequence. At this stage of cassette design, a decision on the selection and monitoring of transformant must be made as well. Therefore, selection or reporter genes (e.g., *gus*, *gfp*, *kan*) should be present within the expression construct [34,35,36].

Advances in the assembly of large DNA fragments, where the exact number and arrangement of transgenes are determined, as well as increase in the efficiency of DNA cloning methods, have contributed significantly to the acceleration of the process of constructing a multigene expression cassette construct [37]. Currently, scientists have at their disposal a large array of kits/systems for implementing the intended strategy of constructing an expression cassette for plant transformation [38,39]. The most commonly used include Golden Gate and derived methods such as MoClo (for chloroplast engineering), Golden Braid, and Green Gate, which are based on the use of type II restriction enzymes [37]. However, the latter feature is not entirely desirable in the case of large multigene constructs, due to numerous recognition sites. To overcome such obstacles, systems based on rare-cutting enzymes have been developed—e.g., the COLORFUL Circuit system (which allows up to five cassettes to be inserted) or the AssemblX system [38,40].

One of the most recent technological innovations in the life science sector is genome editing (GE). This powerful technology has been used to engineer genomes using various editing tools, including zinc-finger nucleases (ZFNs) and transcription activator-like endonucleases (TALENs). The clustered regularly interspaced short palindromic repeats (CRISPR)/ CRISPR-associated9 (Cas9) endonuclease system is also one of the GE’s strategies, which originally evolved prokaryotic organisms as a defence system. The CRISPR/Cas9 system is a very precise tool based on a selective site-directed mutagenesis strategy for RNA-guided genome-editing. This tool exploit is designed to guide RNAs that identify a protospacer adjacent motif (PAM) sequence occurring downstream of the target-DNA [41]. The CRISPR/Cas 9 system was successfully implemented to engineer genomes of various plants, including crops showing resistance to environmental stresses, nutrient content enrichment, and yield improvement [42]. Most recently, the CRISPR/Cas9 strategy was used to edit *DCL2 and DCL4* genes dicer-like in *N. benthamiana* plants. This resulted in plants with a double knock-out of these genes, which consequently affected the increased accumulation of GFP protein and human fibroblast growth factor 1 (FGF1) in comparison with WT and RNA-dependent RNA polymerase *6*-knockout *N. benthamiana* plants [43]. Such results are extremely promising in view of the requirements for increased production of recombinant proteins in plant cells.

**Figure 2:**
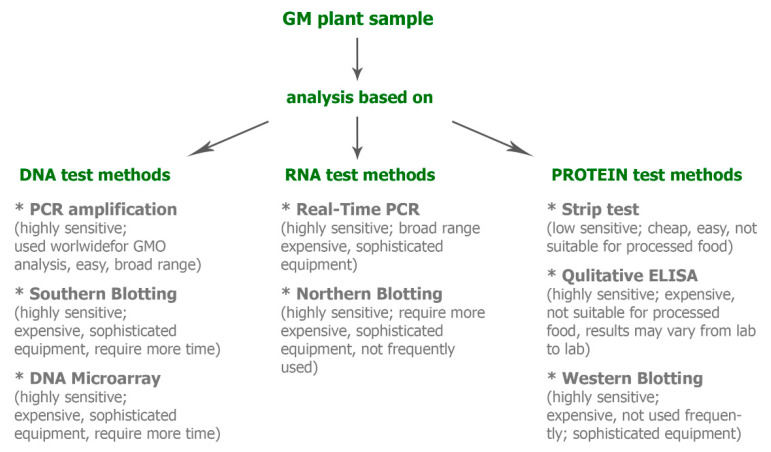
Molecular methods for analysing genetically modified (GM) plants.

The introduction of GM plants or their products onto the market requires the knowledge and monitoring of the presence of individual genetically modified components. Therefore, an indispensable step is to the identify them at the molecular level. A wide variety of methods for this purpose are used (Figure 2).

## 4. Available Strategies for Plant Host Transformation

After the expression cassette is constructed, the recombinant DNA must be introduced into the host cell (here, into the plant cell). Generally, two main routes are used for the production of recombinant proteins in plant systems: stable and transient expression of the transgene. Direct (e.g., particle bombardment) and indirect (bacterium-mediated transformation) methods are used to modify the nuclear genome in order to obtain the stable expression, whilst chloroplast transformation uses only direct methods. In the case of transient expression—providing rapid synthesis of the desired proteins, usually taking a couple of days—viral infection or agroinfection is used [13]. As a result of the stable transgene expression, stable transgenic lines are generated, where a recombinant protein is produced in subsequent generations. Typically, in the early stages of designing a method for the production of the selected recombinant protein, a model plant (e.g., *N. tabacum*, *N. benthamiana*, *A. thaliana*) [27] is selected as the producer. Then, attempts are made to obtain the synthesis of the protein of interest in the target plant, including crops of high economic importance [25,44]. It should be emphasized that the production of a given recombinant protein may not be effective, which may be the consequence of the position effect, gene silencing, or protein degradation, among other things. To overcome these obstacles, the production of recombinant proteins is directed to cellular compartments including chloroplast, endoplasmic reticulum, vacuole, cytosol, or apoplast [45,46]. This sorting of proteins is accomplished by adding appropriate signal sequences (e.g., KDEL) to the gene construct [47]. In addition, some differences in the pattern of glycosylated proteins create a problem with the quality of the therapeutic proteins of plant origin (Tables 2 and 3). The consequence of this may be the risk of allergies and even anaphylactic shock after taking such a drug [48]. Several approaches have been developed to overcome this obstacle, including (i) placing human glycosyltransferases in the plant genome to modify protein glycosylation pathways [49,50]; (ii) knocking out or knocking down specific plant glycosyltransferases [51,52]; (iii) accumulating target proteins in cell compartments where plant-specific glycosylation does not take place (endoplasmic reticulum) or where proteins are not glycosylated (cytosol or plastids) [45,46].

In addition to modifying the nuclear genome, the plastid genome is also extensively studied in respect of the stable expression of recombinant proteins. Two routes are mainly used for the transfer of the chloroplast genome: the biolistic method and transfer via polyethylene glycol (PEG). The first strategy is to deliver coated DNA molecules using a gene shotgun. This method can be applied to any plant species by adjusting biolistic bombardment parameters, including chamber vacuum pressure, distance to plant tissue, and particle size [53]. The second strategy is to place the protoplasts in the presence of PEG, which facilitates the uptake of naked DNA by the cells [54]. More recently, a new approach was used by Kwak et al. [55], where nanoparticles consisting of chitosan-complexed single- walled carbon nanotubes (CS-SWNTs) were used to deliver DNA to chloroplasts. As a result of electrostatic interactions between negatively charged DNA and positively charged nanoparticles, conjugates are formed that can easily penetrate into mesophyll cells through the pores of the stomata. In order to obtain maximum efficiency of transformation, as in the case of nuclear transformation, it is important to choose the right promoter, regulatory sequences, and insertion sites. Promoters commonly used in plastid transformation are *Nicotiana tabacum* promoter from the plastid encoded photosystem II protein D1 precursor (*psbA)*; *N. tabacum* promoter from the plastid rRNA operon(*rrn*); or *Chlamydomonas reinhardtii/N. tabacum* chloroplast RuBisCo large subunit (*rbcL*) and the untranslated UTR regions: *ggagg*, T7g10, *rbcL*, *psbA*, *atpB*; 3 UTRs: *psbA*, *rps16*, *rbcL*, *petD*; *trnl*/*trnA*, *rbcL*/*accD*, *trnfM*-*trnG*, *trnV*/*rps12*, *trnN*-*trnR*, *ycf3*-*trnS* [54]. Transplastomic technology has many advantages over the modification of the nuclear genome, including the high level of transgene expression (from 46% to more than 75% of total soluble protein) resulting from a large number of copies of the chloroplast genome (about 10,000 copies of the chloroplast genome in a single cell), coverage for the simultaneous expression of multiple transgenes, ability to properly assemble and produce disulfide bridges, the lack of silencing effect due to site-specific transgene integration into the plastid genome, and the possibility of relatively easy elimination of marker genes. Moreover, the vast majority of crops do not contain plastids in pollen; therefore, the transgene will not spread through it [51,52,53]. To date, the engineering of the chloroplast genome of edible leafy crops (e.g., *Lactuca sativa* or *Brassica*) has been of great interest due to the crops’ use as a potential source of edible vaccines, and the chloroplast transformation protocols of many important crops (e.g., carrot, cotton, eggplant, potato, tomato, soybeans) have been successfully established [54,56]. 

An alternative approach for obtaining transgenic plants that produce high levels of proteins in a few days is transient expression. It is based on the use of *Agrobacterium* (agroinfection/agroinfiltration) or a properly engineered plant virus system (e.g., Bromoviruses, Comoviruses, Gemniviruses, Potexviruses, Potyviruses, and Tobamoviruses) to introduce a transgene. In addition, this technology does not generate high costs due to its simplicity, and it has no requirements for expensive equipment. Leaf blades of ex vitro growing plants are most often infiltrated using a syringe, spraying method (agrospray), or vacuum infiltration. Additionally, in order to enhance transformation efficiency, the infiltration medium is enriched with surfactants [35,45]. 

A significant breakthrough in the improvement in transient expression was the development of the MagnICON^®^(Icon Genetics GmbH, Halle, Germany) system, based on the deconstructed viral vector system, which allowed for a very fast accumulation of heterologous proteins in an extremely short time [45]. The MagnICON^®^ system combines the advantages of three biological systems: (i) exploitation of *Agrobacterium* as a viral vector provider, which eliminates the need to produce separate RNA vectors; (ii) rapid and efficient levels of viral RNA expression (without the risk of creating infectious particles); (iii) the ability to conduct eukaryotic post-translation modifications of complex proteins. Taking into account these advantages of magnifection, it is fully justified to state that it is a biologically safe, low-cost, and fast technology [13]. 

Since then, the transient expression system has been constantly refined and developed. To this day, it has been used in the production of various therapeutic proteins (e.g., monoclonal antibodies, vaccine subunits, growth factors, cytokines) and diagnostic and industrial enzymes. The application nature of this system for the production of recombinant proteins was summarized by Gleba et al. [13]. The plants transformed through the discussed system include *N. benthamiana*, eggplant, hot peppers, lettuce, melons, tomatoes, and orchids [57]. Taking into account the benefits of the transient expression system in plants, it can be an excellent response to the demands of the pharmaceutical market, which is to meet the need for the production of therapeutic proteins with high efficiency in a short time. This is extremely important, especially in the light of recent world events, including the COVID-19 pandemic. An example is the study by Diego-Martin et al. [58], where transient expression in *N. benthamiana* was used to produce six recombinant anti-SARS-CoV-2 monoclonal antibodies at laboratory scale and a pilot upscaling of two of those six antibodies.

## 5. Downstream Processing Strategies and Purifications

The synthesis of recombinant proteins in plants at a high level is quite a challenge, but so is their recovery from plant material. This stage is extremely important, and moreover, generates significant costs (80–90%) in the entire production process [1]. 

### 5.1. Protein Extraction

One major challenge is the effective and comprehensive extraction of proteins from plant tissues. This stage is critical, as it determines the quality as well as the concentration of the target protein. It should be emphasized that plant tissues are very difficult to destroy for full extraction of intracellular products, due to the high content of lignohemicellulose compounds in their cell walls. Widely used intracellular extraction methods are not applicable to plants and fungi. Standard methods of protein extraction are based on the lysis of cells by mechanical (homogenization) or chemical methods (osmotic shock, enzymatic digestion). Next, the resulting biomass is suspended in an appropriate buffer, which provides appropriate pH and salinity, among other factors. At this stage, it is necessary to add protease inhibitors to protect the target proteins against proteolysis [1]. In the next step, the obtained mixture is purified. The most commonly used extraction method from plant tissues is CTAB or SDS-based buffers [35,59]. Commercially available kits such as Qiagen DNeasy Mericon Kit or Qiagen DNeasy Plant Mini Kit (Qiagen, Hilden, Germany) can also be used [59]. 

A common approach used with plant expression systems is to use modifications so that recombinant proteins accumulate in various plant tissues (e.g., fruit, seeds, leaves, roots). The disadvantage of such a strategy, apart from the high cost of protein recovery, is the necessity to harvest the plants, which means that it is not possible to continuously synthesize target proteins. Therefore, an interesting solution seems to be production with the use of a strategy based on the system of secretion of proteins to the medium. The secretion-based system is successfully used in bacterial or mammalian cell cultures [60]. This technology has also been implemented in plant cell suspensions [61] or hairy root cultures [62] and whole plants (hydroponic cultures) [63]. Nevertheless, as mentioned earlier, plant cells secrete many of host proteins into the medium, including proteases that degrade recombinant proteins. Hence, the process of their recovery and purification poses a significant challenge.

Guttation is another non-destructive way that allows for the recovery of recombinant proteins produced by plants. It is the natural plant mechanism for removing water together with various dissolved substances. It is assumed that by regulating the physicochemical parameters (e.g., aeration, temperature, light, humidity, nutrition, phytohormones supplementation), the intensity of the guttation process and thus the recovery of target proteins can be influenced [64]. Despite the enormous potential of this technique, it requires further research and improvements.

In many systems based on the production of recombinant proteins by whole plants, modifications are made so that the target proteins remain in selected cell compartments or are secreted into the apoplast. One of the known techniques for recovering soluble proteins from the apoplast is the method of vacuum infiltration–centrifugation. In this procedure, omitting the step of homogenization of plant tissues in order to recover proteins significantly reduces the costs of the entire process [46].

### 5.2. Clarification

Before the mixture obtained after extraction is properly purified, it undergoes a clarification process. This is a necessary step due to the fact that as the tissue disrupts, numerous contaminants are released, including chlorophyll, polysaccharides, soluble proteins, RNA, DNA, and phenols, with the latter impurities being especially disadvantageous because they can be responsible for structural or conformational changes in target proteins [65]. Usually, to obtain a well-clarified extract, filtration and centrifugation are used together, which significantly increases the costs of downstream processing. The filters are disposable, and often the clarification process requires the use of several filtrations using various filters dedicated to different pollutants [27]. Additionally, flocculants are used to increase the filtration efficiency. They are high molecular weight polymers carrying a strong positive or negative charge. Charged flocculants facilitate the separation of molecules from fluids by increasing their aggregation. However, it should be emphasized that the behaviour of polymers is determined by many factors (e.g., molecular weight and charge density, pH, conductivity of the medium), which may also significantly affect the degree of recombinant protein recovery. Therefore, appropriate selection of flocculants is recommended in order to optimize the entire process. Among the flocculants frequently used for the precipitation of crude particles from plant extracts are the following: Praestol, Magnafloc, Sedipur (Solenis LLC, Wilmington, DE 19803, United States), Lupamin, Polymin, Lupasol, Catiofast GM (BASF AG, Ludwigshafen, Germany), ZETAG (Brenntag NV, Deerlijk, Belgium) [66].

### 5.3. Protein Purification

Despite the fact that the technology of producing therapeutic proteins in plants has changed and developed since the idea of molecular farming was born, the purification stage is still the most important hindrance. Generally, recombinant proteins (synthesized in any system) intended for human or animal therapy must be of a high degree of purity. Hence, the purification procedure is focused on obtaining proteins free from any contamination, while maintaining their correct chemical structure and biological function [67,68]. The cost of protein purification accounts for 45% to 92% of the overall manufacturing process [1]. However, it is difficult to competently compare both the purification and total production cost of 1 g of recombinant protein since the values found in the literature are often based on different calculation methods and also depend on protein complexity, etc. According to the report cited by Schillberg and Finer [27], the cost of 1 g of a human antibody produced from transgenic tobacco tissues was estimated at EUR 1137, where, as it is important to note, the downstream processing accounts for 84% of the total cost. By contrast, techno-economic models allow for the production of 1 g of purified algae-derived lectin that the costs only USD 106 [69]. In this case, the downstream processing cost of algae-derived lectin is estimated at 50% of the total cost. The difference is significant and assigned mainly to the cost of the first antibody purification step. However, it must be emphasized that the aforementioned techno-economic models did not estimate capital equipment, total capital investment costs, local taxes, and other expenses of this kind. Nevertheless, even the data from the first example are extremely promising, as they may suggest that due to the cost of protein production in plants, they can compete with other frequently used systems for the production of therapeutic proteins, such as mammalian or bacterial cells. The lower up-front investment in the case of plants, along with purification methods still being optimized on the one hand, and the relatively low risk of producing a misfolded protein on the other hand, seem to confirm the plant potential.

When planning plant-derived protein purification strategies, other factors such as high recovery, ease, and repeatability should be considered in addition to economic considerations. In order to meet the strict purity requirements of the biopharmaceutical industry in the production of therapeutics, chromatography is used during purification of the protein product [67]. Chromatography is undeniably the method of choice, although it is mostly used for small-scale purification of recombinant proteins. Depending on the specificity of the proteins isolated (e.g., size, hydrophobicity, charge, etc.), various types of chromatography are used, including affinity chromatography (AF), immobilized metal affinity chromatography (MAC) or ion exchange chromatography (IEX), hydrophobic interaction chromatography (HIC), reverse-phase chromatography (RPC), and size exclusion chromatography (SEC). These methods were discussed in detail by Owczarek et al. [1]. Due to the cost and complex nature of the above-mentioned methods, their application on a large scale poses a major challenge. Hence, non-chromatographic methods are currently enjoying great interest [70,71].

#### 5.3.1. Elastin-Like Polypeptides

Elastin-like polypeptides (ELPs) are biopolymers that contain repeats of a hydrophobic pentapeptide (Val-Pro-Gly-Xaa-Gly), where Xaa (guest residue) can be any amino acid except proline because it deprives the elastin-like polypeptides of specific features. ELPs possess the ability of a so-called reverse phase transition—i.e., they pass from a soluble form in solutions to an insoluble form. Interestingly, this phenomenon is reversible and closely correlated with the phase transition temperature (Tt) for the biopolymers being discussed here. Due to their feature, ELPs have become an attractive tool for quick and easy purification of recombinant proteins. The resulting insoluble protein–ELP aggregates can be easily separated from impurities and collected by centrifugation [70]. ELPs can be bound to inteins to release the recombinant protein from the ELP complex. Inteins (protein-splicing elements) are capable of self-cleavage as a result of changes in the pH of the solution under the influence of thiol residues [72]. ELPs in combination with inteins offer an effective and inexpensive system for the purification of recombinant proteins produced in plants. Furthermore, the use of ELPs in fusion proteins positively influences recombinant protein expression [73].

#### 5.3.2. Oleosin Fusion Expression System

Oleosins are amphipathic proteins naturally occurring in plants. They prevent oil bodies from coalescing during seed maturation. Their presence has also been found in pollen. Due to their unique physicochemical properties, oleosins have been applied in a number of procedures, including protein purification [74]. It should be emphasized that these oleosins can be very easily separated from other cellular components by centrifugation [75]. Oleosin::recombinant protein fusion is found in oil bodies in transgenic seeds. Generally, to obtain the desired product from the seed, the latter must be ground and treated with an appropriate aqueous buffer and then subjected to a series of centrifugations and washes with the buffer, changing the salts and pH of the environment. The oleosin::recombinant protein fusion site is designed to include a unique cleavage site to allow separation of the desired protein from the oleosin that will remain in the oil bodies. Finally, the oil bodies together with the oleosin are removed by centrifugation, while the recombinant protein undergoes further processing [74]. Due to the simplicity of this purification method, as well as to the fact that it is not time-consuming compared with more sophisticated chromatography, it was patented, in many cases with a pharmaceutical application [74].

## 6. Production of Pharmaceutical Proteins in Crop Plants

Established in the 1980s, the concept of using plants to produce therapeutic and non-pharmaceutical proteins has been intensively developed during the last decade, and the results have been well documented in numerous publications. Many reports have unequivocally proved that plant systems are great candidates for the production of biopharmaceuticals or potential vaccines for human or animals (Table 2).

Usually, the first-to-try systems are model plants, which, as in the case of tobacco leaves, offer desired biomass level. Nevertheless, the great economic importance of many crop species constituting a human diet led to the development of transformation and regeneration procedures for them. So far, the possibility of producing recombinant proteins in different fruits and vegetables (e.g., strawberries, bananas, potatoes, tomatoes, lettuce, spinach, rice, safflowers, barley) has been proven [99,100,101,102]. Significant attention was focused on leafy plants such as alfalfa, lettuce, or clover, which could serve as edible vaccine producers, thus eliminating unpleasant injections and, above all, the purification phase, which is associated with a reduction in overall production costs [103,104]. Plant expression platforms have also been tested for the production of recombinant virus-like particles (VLPs), which is an interesting vaccine strategy. These large-scale studies focused on widespread viruses, including foot-and-mouth disease virus, norovirus, influenza virus, poliovirus, and rotavirus [105,106,107,108,109]. The tremendous advances in the development of virus-based transient expression have overcome two major problems: initially low antigen accumulation and long production times. It has been shown that a high level (1–2.3 mg/g LFW) of expression of norovirus VLPs in plants can be achieved using an appropriately modified geminiviral vector [106]. 

Huang et al. [110] reported the production of recombinant human alpha-1-antitrypsin (rAAT) glycoprotein, exploiting a chemically inducible cucumber mosaic virus (CMV) viral amplicon expression system in transgenic *N. benthamiana* cell culture. By optimizing the production process in a semicontinuous bioreactor, a 25-fold increase in the production of extracellular functional rAAT (603 mg/L) was obtained.

### Examples of Clinical Trials and Commercialization of Plant Recombinant Proteins

By 2021, more than twenty studies on therapeutic proteins for humans or animals, produced on the basis of the crop plant system, had obtained the status of preclinical research or clinical trials [4,111]. In many instances, *N. tobacco*, *N. benthamiana*, or *A. thaliana* are the target species for the production of therapeutic proteins undergoing clinical trials [1]. Nevertheless, more and more often, the producers of the tested proteins are food crops (Table 3). One of the notable cases is the production of recombinant glucocerebrosidase (prGCD) (an enzyme protein) in a suspension of carrot cells (ProCellEx™, Protalix Biotherapeutics, Carmiel, Israel). This product, sold under the trade name Elelyso™ (Protalix Biotherapeutics, Carmiel, Israel), was the first system of this kind approved by the FDA in 2012 and is used in enzyme replacement therapy to treat patients with Gaucher disease. The USDA-approved oral veterinary vaccine against Newcastle disease obtained from corn is another example [14]. 

Human insulin was one of the first pharmaceutical proteins that was produced in bacteria cells using recDNA technology. Over time, it transpired that fully functional insulin can be successfully produced in the seeds of a plant (e.g., *A. thaliana*, *Zea mays*) [116,124]. Using the oleosin fusion strategy, the accumulation of the described protein was achieved at the level of over 0.1% of TSP (total soluble protein) [124]. The above-mentioned solution was used by SemBioSys Genetics Inc. (Calgary, AB, Canada) for the production of insulin on an industrial scale. Currently, insulin obtained from safflower has passed the second and third phase of clinical trials, and the results are very promising [102].

The production of therapeutic proteins in plant systems has attracted great attention due to the lower cost of the plant material production. It was not only economic considerations that made plants a suitable production system but also the possibility of rapidly increasing the scale of target protein manufacturing. Furthermore, the pharmaceutical industry should react very quickly to the needs of the market, especially in situations where we deal with a pandemic and it is necessary to deliver a large number of vaccines in a short time. The use of the MagnICON® technology in the leaves of *N. benthamiana* to produce ZMapp, an experimental drug which is the mixture of three monoclonal antibodies, is such an outstanding achievement. This drug was used to treat people during the 2014 Ebola outbreak in West Africa, without having previously undergone any clinical trials assessing its potential risks. Clinical trials (phases 1 and 2) for ZMapp were conducted in 2015 [3]. The study found that for those who received Zmapp, the risk of death was 40%. Although the results were statistically insignificant and did not indicate whether ZMapp works, the drug was shown to be safe and well tolerated [125].

Arntzten et al. pioneered the implementation of the concept of edible vaccines. They demonstrated the possibility of producing antigens (HBsAg) against hepatitis B (HBV) in tobacco leaves. Then, they used potato tubers to produce these antigens, which were orally administrated to humans. According to their findings, increased systemic and mucosal immunity was noted in 62.5% of volunteers after consuming three doses of transgenic potato tuber (100 g per dose) [80]. These results quite clearly indicated the potential of a plant-based vaccine in the global prevention system against hepatitis B.

The outbreak of the COVID-19 epidemic has sparked a race among biotech companies to develop an effective vaccine against SARS-CoV-2. Researchers at the University of California San Diego have proposed a cowpea-produced vaccine that is in the preclinical testing phase [17]. This race was joined by Medicago, which developed CoVLP, a potential vaccine produced in *N. benthamiana* leaves, which is currently in phase 3 of clinical trials. Research results have proven that the CoVLP vaccine is safe and effective (it generates 10 times higher immune response compared with plasma of convalescent patients) [18]. Kentucky Bioprocessing has developed a vaccine (KBP)-201 that has passed phase 1 and 2 clinical trials [126]. Examples of plant made biopharmaceuticals at various stages of development and implementation on the market are collected in Table 4.

## 7. Conclusions

Considering the market capacity and how many pharmaceutical proteins are being introduced into it, plant systems as producers remain a niche platform. Nevertheless, due to the whole range of unique advantages (e.g., eukaryotic biosynthetic pathways, lack of contamination with viral pathogens, lack of contamination with endotoxins, relatively low production costs, etc.), plants are a competitive expression system compared with conventional ones (e.g., bacteria, yeast, or human cells) for the production of various recombinant proteins (pharmaceutical and non-pharmaceutical). The production of therapeutic proteins in plants using a transient expression system, allowing the product to be obtained in just a few days, is an undoubted advantage. 

Over the course of several decades, various strategies for the production of specific pharmaceutical proteins for both humans and animals in plants have been developed and refined. In many cases, this resulted in obtaining patents, in clinical trials, and as a result, commercialization. Nevertheless, the path from the concept of the production of a therapeutic protein in plant systems to its implementation on the market is extremely long and difficult. This is due to many reasons. One of the main barriers to the implementation of molecular farming on an industrial scale, especially in the field of pharmaceutical production, is the fear of contamination of the natural ecosystem with pharmaceuticals and, consequently, fear of contamination of the food chain. This is especially true when edible crops (e.g., potato, tomato, rice, corn) are used as expression platforms for the production of recombinant proteins. Due to the lack of information, the public is concerned that drugs obtained in plant systems may pose a health risk by triggering allergic reactions. It should be emphasized, however, that proteins manufactured in plants are subject to the same quality control standards as pharmaceuticals produced in bacterial, animal or yeast systems. Biopharmaceuticals produced in the world—in particular, those approved by the WHO, the EU, or the USA—must meet certain requirements referred to as Good Manufacturing Practice (GMP), Good Laboratory Practice (GLP), and finally Good Clinical Practice (GCP) [1]. The biopharmaceutical production process is strictly controlled at every stage, and what is more, the finished product is tested for toxicity or the presence of viral contaminants. Sahoo et al. [28] provided a very detailed overview of the production and approval of biopharmaceuticals. They pointed to the need to unify the regulations governing the production of biodrugs, which would greatly facilitate their introduction onto the market and sale in various countries. Another important aspect that slows down the wider use of molecular farming in the production of biopharmaceuticals is the fact that the industry prefers to rely on known and well-established technologies. Often, proprietary technologies that have been developed limit the freedom of action and narrow the circle of potential industrial business partners. The Pharma-Factory project [27] was created from EU funds for large-scale commercial use of molecular farming. The main assumption of this project is to support innovation in the field of molecular farming and, above all, to remove technical regulations that hinder public acceptance and exit from the laboratory research phase to the market. Considering the enormous potential of plants as producers of therapeutic proteins, it seems reasonable that, apart from raising public awareness of this topic, there is a great need to support research groups and the pharmaceutical industry in their pursuit of the commercialization of as many necessary plant-derived drugs as possible. This is extremely significant in order to improve the quality and comfort of human life, which is of particular importance in the light of a crisis situation such as a pandemic.

## Figures and Tables

**Table 1:** Crucial facts in the development of plant-made pharmaceuticals.

Year	Achievement	Bioreactor	Stage of Drug Development	References
1998	Production of secretory antibody IgG-IgA against tooth decay caused by *Streptococcus mutans*	tobacco plants	approved; brand name–(CaroRX^®^, Planet Biotechnology INC, Hayward, CA, USA)	Juarez et al. [9]
1998	First plant-made vaccine (LTB)	potato	clinical trial (phase 1) oral administration route	Tacket et al. [10]
2004, 2005, 2014	Establishment and development of a new strategy (magnifection) for increasing recombinant protein production in plant platform	*Nicotiana benthamiana*	several pharmaceuticals, e.g., vaccines for Non-Hodgkin’s lymphoma completed clinical trials in 2013	Gleba et al. [11,12,13]
2006	Newcastle disease (NDV) vaccine licensed for veterinary use	maize	licensed for veterinary use	Guerrero-Andrade et al. [14]
2008	Plants have been shown to be a fast and efficient system for producing an influenza vaccine	*N. benthamiana*	laboratory/pre-clinical stage	D’Aoustet et al. [15]
2012	Production of taliglucerase alfa for Gaucher’s Disease	carrot cells	approved by FDA ELELYSO™	Yao et al. [3]
2015	Clinical trial (I phase) of plant-made vaccine against cancer (follicular lymphoma) demonstrated its safety	tobacco plants	clinical trial (phase 1)	Tuse et al. [16]
2015	Obtaining experimental drug (comprising three chimeric monoclonal antibodies) for Ebola virus	tobacco plants	clinical trials phase 1 and 2; in 2015 ZMapp was granted fast-track status by the FDA	Yao et al. [3]
2015	Production of immuoadhesin (DPP4-Fc) which prevents the MERS-CoV from infecting lung cells	tobacco plants	pre-clinical phase	Yao et al. [3]
2021	Production of potential vaccine against SARS-CoV-2	cowpea	pre-clinical	Ortega-Rivera et al. [17]
2021	Production of CoVLP—potential vaccine against COVID-19	*N. benthamiana*	clinical trials (phase 3)	Gobeil et al. [18]

COVID-19, coronavirus disease; NDV, Newcastle disease; FDA, Food and Drug Administration; MERS-CoV, Middle East respiratory syndrome coronavirus; SARS-CoV-2, severe acute respiratory syndrome coronavirus 2.

**Table 2:** Selected therapeutic proteins (potential vaccine candidates and antibodies) produced in edible crop plants.

Recombinant Protein	Disease	Plant	Expression Level	Reference
VP60 structural protein	Rabbit haemorrhagic disease virus (RHDV)	potato	0.3% of TSP	Castanton et al. [76]
Hemagglutinin protein of rinderpest virus	Rinderpest virus (RPV)	peanut	0.2–1.3% of TSP	Khandelwal et al. [77]
Spike (S) protein of transmissible gastroenteritis virus	Transmissible gastroenteritis virus (TGEV)	corn	13 mg/kg FW	Lamphear et al. [78]
Spike (S) protein of	Infectious bronchitis virus (IBV)	potato	2.39–2.53 µg/g FW	Zhou et al. [79]
Hepatitis B virus surface antigen	Hepatitis B virus (HBV)	potato	8.5 µg/g FW	Thanavala et al. [80]
Fusion (F) protein of Newcastle disease virus	Newcastle disease virus (NDV)	corn	3.0% of TSP	Guerrero-Andrade et al. [14]
F4 fimbrial adhesion FaeG	Enterotoxigenic *E. coli*	alfalfa	1.0% of TSP	Joensuu et al. [81]
Recombinant Norwalk virus (rNV) capsid protein	Norwalk virus (NV)	tomato; potato	0.4 g freeze-dried tomato fruit containing 64 µg rNV (40 g VLPs); 1 g freeze-dried potato tuber containing 120 µg rNV (90 µg VLPs)	Zhang et al. [82]
Heat-labile toxin B subunit (LTB)	Enterotoxigenic *E. coli*	soybean	2.4% of TSP	Moravec et al. [83]
VP2 structural protein	Infectious bursal disease virus (IBDV)	rice	40.21 µg/g FW	Wu et al. [84]
Heat-labile toxin B subunit (LTB)	Enterotoxigenic *E. coli*	carrot	3.0% of TSP	Rosales-Mendosa et al. [85]
VP1 structural protein	Foot and mouth disease virus (FMDV)	legume	0.1–0.5% of TSP	Wang et al. [86]
Japanese encephalitis virus (JEV) envelope protein E	Japanese encephalitis virus (JEV)	Japonica rice	1.1–1.9 µg/g FW	Wang et al. [87]
UreB subunit	*Helicobacter pylori*	carrot	25 mµg/g	Zhang et al. [88]
MLC chimeric recombinant gene	Vivax malaria	rapeseed	N/A	Lee et al. [89]
E2 structural protein	Bovine viral diarrhoea virus (BVDV)	alfalfa	1 µg/g FW	Perez Aguirreburualde et al. [90]
scFvT84.66	Cancer (tumour marker)	rice	3.8 µg/g FW	Torres et al. [91]
scFvT84.66	Cancer (tumour marker)	wheat and rice	30 µg/g FW	Stöger et al. [92]
HIV-1 p24 antigen	HIV	carrot	62 ng/g FW	Lindh et al. [47]
6D8	Ebola virus	lettuce	0.23–0.27 µg/g	Lai et al. [93]
Protective antigen (PA) gene	Anthrax	Indian mustard	NR	Gorantala et al. [94]
Altered peptide ligands of type II collagen rheumatoid arthritis	Rheumatoid arthritis	rice	NR	Iizuka et al. [95]
Recombinant HCV core protein	Chronic liv er disease	rapeseed	0.05% of TSP	Mohammadzadeh et al. [96]
Fusion protein CFP10-ESAT6-dIFN	Tuberculosis	carrot	28.140 μg of TSP	Permyakova et al. [97]
2G12	Human immunodeficiency virus (HIV)	rice	46.4 µg/g DSW	Vamvaka et al. [98]
Epithelial cell adhesion molecule EpCAM–IgM Fc	Cancer	Chinese cabbage	NR	Lee et al. [99]

TSP, total soluble protein; FW, fresh weight; DSW, dry seed weight; NR, not reported; VLPs, virus-like particles.

**Table 3:** Different recombinant proteins produced in crop plants.

Recombinant Protein	Plant	Expression Level	Plants Platform	Reference
Human serum albumin	potato	0.25 µg/mg (0.02% of TSP)	leaf; cell culture	Sijmons et al. [112]
α 1-antitrypsin	rice	4.6–5.7 mg/g dry cell	cell culture	Terashima et al. [113]
Aprotinin	corn	0.069% of TESP total extractable seed protein	seeds	Zhong et al. [114]
Human basic fibroblast growth factor (bFGF)	soybean	2.3% of TSP	seeds	Ding et al. [115]
Human recombinant proinsulin	corn	18.87 mg/L (0.42% of TSP)	seeds (endosperm)	Farinas et al. [116]
α 1-antitrypsin	tomato	1.55% of TSP	shoots	Agarwal et al. [117]
Human interferon gamma	rapeseed	NR	seeds	Bagheri et al. [118]
Staphylokinase	potato	NR	shoots	Gerszberg et al. [36]
Lumbrokinase	sunflower	5.1 g/kg	seeds	Guan et al. [119]
Proinsulin	tomato	NR	shoots	Soltanmohammadi et al. [120]
Human proinsulin	strawberry	0.15% TSP	shoots and roots	Tavizi et al. [121]
Human gastric lipase (hGl)	turnip	11 mg/L	hairy root	Ele Ekouna et al. [122]
Human alpha-L-iduronidase (IDUA)	rapeseed	NR	hairy root	Cardon et al. [60]
L-asparaginase II (*ansB*) gene	potato	NR	hairy root	Mohammadi et al. [123]

TESP, total extractable seed protein; TSP, total soluble protein; NR, not reported.

**Table 4:** Examples of food crop PMPs at miscellaneous stages of development.

Crop	Product	Disease/Purpose	Development Stage/Study	Company	References
Banana (leaf)	PRRSV (envelop glycoprotein)	Porcine reproductive and respiratory syndrome virus	Development	National Taiwan University, Taiwan, Republic of China	Chan et al. [101]
Barley (seed)	Human epidermal growth factor; Human growth hormone	burn treatment; deficiency treatment	Commercialisation	ORF, SifCosmetics	Park et al. [102]
Carrot (cells suspension)	Alpha-galactosidase (PRX-102)	Fabry disease	Phase 3	Protalix Bio-therapeutics (Israel)	Schiffmann et al. [127]
Carrot (cells suspension)	Acetylocholesterase (PRX105)	Biodefense	Phase 1	Protalix Bio-therapeutics (Israel)	Atsmon et al. [128]
Carrot	HIV-1 p24	Immunodeficiency syndrome	Development	Örebro Life Science Center, Örebro University,	Lindh et al. [129]
Carrot (cells suspension)	Glucocerebrosidase (Elelyso)	Gaucher’s disease	Approved by FDA 2012—on market	Protalix Bio-therapeutics (Israel)	Zimran et al. [130] Owczarek et al. [1]
Chinese cabbage (seed)	Epithelial cell adhesion molecule (EpCAM)	Potential anticancer vaccine candidate	Development	National Institute of Horticultural and Herbal Science, Rural Development Administration, Korea/ Chung-Ang University, Seoul, Korea	Lee et al. [37]
Corn	Meripase®	Cystic fibrosis	Commercialisation	Meristem Therapeutics (France)	Gayatonde et al. [131]
Corn	Avicidin (antibodies)	Colorectal cancer	Phase 2	NeoRX/Monsanto (USA)	Edgue et al. [132]
Indian mustard	Protective antigen	Potential anthrax vaccine	Development	School of Biotechnology, Jawaharlal Nehru University, New Delhi, India	Gorantala et al. [94]
Lettuce	MV-H protein	Measles Virus	NA	Monash University, Melbourne/ MacFarlane Burnet Institute for Medical Research and Public Health	Webster et al. [100]
Potato	Albumin	Diagnostic	Commercialisation	Synthon	Park et al. [102]
Potato Tomato	Norwalk virus capsid protein	Norovirus vaccine	Phase 1; Pre-clinical	Arntzten team, Arizona State University; Biodesign Institute and School of Life Sciences (USA)	Tacket et al. [133] Huang et al. [134]
Tomato (fruit)	IgA	Hand, foot, and mouth disease (HFMD) Rotavirus	Development	National Taiwan University, Taipei	Chen et al. [135]
Rice (seed)	Type II collagen (CII256-271 and APL6)	Rheumatoid arthritis	Development	University of Tsukuba, Tsukuba, Japan/ National Institute of Agrobiological Sciences, Tsukuba, Japan	Iizuka et al. [95]
Rice (seed)	Alpha subunit of soybean	Hypercholesterolemia	Development	Kyoto University, Uji, Kyoto, Japan/ Gifu University, Gifu, Japan	Cabanos et al. [136]
Spinach	Glycol protein	Hepatitis B	Phase 1	Institute of Biotechnology and Antibiotics (Poland)	Chen and Lai [137]
Spinach	Rabies lycoprotein	Vaccine	Phase 1	Yusibow group, Fraunhofer USA	Yusibow et al. [138]
Strawberry (fruits)	Canine interferon α (oral vaccine)	Canine periodontal disease (veterinary purpose)	Commercialisation	NAIST	Park et al. [102]

GRAS, generally recognized as safe; NA, non-available; NAIST, National Institute of Agrobiological Sciences, Tsukuba.
